# Identity, Prevalence, and Pathogenicity of Entomopathogenic Fungi Infecting Invasive *Polistes* (Vespidae: Polistinae) Paper Wasps in New Zealand

**DOI:** 10.3390/insects13100922

**Published:** 2022-10-12

**Authors:** Aiden Reason, Mariana Bulgarella, Philip J. Lester

**Affiliations:** School of Biological Sciences, Victoria University of Wellington, P.O. Box 600, Wellington 6140, New Zealand

**Keywords:** *Beauveria*, entomopathogenic fungi, *Hirsutella*, microbial pathogenicity, *Ophiocordyceps*, *Polistes chinensis*

## Abstract

**Simple Summary:**

The Asian paper wasp *Polistes chinensis* is an invasive species in New Zealand and South-East Australia. This species threatens native invertebrate communities through predation and potentially competition for resources. During an ecological field study of *P. chinensis* in New Zealand, we discovered wasps that were infected with and were being killed by two species of pathogenic fungi. In the interest of investigating these fungi as potential biological control agents, our aim in this study was to identify the two fungal species using morphological and molecular methods, determine their natural prevalence in the studied paper wasp population, and test their infective potential to hosts in a controlled environment. We successfully identified the fungi species as *Beauveria malawiensis*, a generalist insect pathogen, and *Ophiocordyceps humbertii*, a specialist of social wasps. These are the first records of these species infecting paper wasps in New Zealand. We estimate that they produce infections in approximately 3.3% of wild *P. chinensis* colonies in our study site. In our infection assays, we passively exposed healthy wasp colonies to asexual fungal spores, which resulted in successful infections of *B. malawiensis*, and significantly increased wasp mortality rates.

**Abstract:**

Two species of entomogenous fungi were discovered infecting the invasive paper wasp *Polistes chinensis* during an ecological study on Farewell Spit, New Zealand. We sequenced two nuclear ribosomal RDNA genes, the internal transcribed spacer (ITS) and the small ribosomal subunit 18S, and one protein-coding gene, the translation elongation factor 1-alpha (*ef1 α*). Combining sequence information with morphological examination, we identified these species as *Beauveria malawiensis* and *Ophiocordyceps humbertii*. We estimated that these fungi produce infection in approximately 3.3% of colonies in our study population. In bioassays, we successfully infected *P. chinensis* individuals from healthy colonies with *B. malawiensis*, with significant effects on adult mortality. This is the first record of both *B. malawiensis* and *O. humbertii* from Polistine hosts in New Zealand, and the first investigation into disease causality by these pathogens in *P. chinensis*. Our findings may contribute to the future development of biological control agents for paper wasps in New Zealand and elsewhere around the world.

## 1. Introduction

The concept of microbial pathogenicity and confirmation of disease causation, first introduced as Koch’s postulates in 1855, has been adapted many times for application in various disciplines [1,2,3,4,5]. The establishment of causal links between fungal species and their infection of hosts is a relevant area of research for improved understanding of the evolution of microbial pathogenicity and host immunity [6,7,8,9], as well as for developing biological control methods for invasive species [10,11,12,13,14]. The pathogenicity of entomogenous fungi to social wasps has remained relatively unexplored compared to fungal associations with other insect groups, however, several studies have shown that there may be potential in these fungi for use in the control of pestiferous wasps [11,12,14].

Entomogenous fungi are important natural regulators of insect populations and are among the most abundant entomopathogens in the world [15,16]. They are a diverse group with members across four fungal phyla but are largely concentrated in the order Hypocreales of Ascomycota [16,17,18]. Entomogenous fungi are unique as Arthropod pathogens in their infection mechanisms, typically entering their host through direct contact with the insect cuticle [19,20,21]. These fungi produce specialised exocellular enzymes including proteases and chitinases that degrade the host cuticle, enabling the penetration of germ tubes or appressorial hyphae through the cuticle and epithelium [19,21,22]. Toxic secondary metabolites then facilitate invasion and colonisation of the host’s haemolymph [23,24]. However, several fungal species may also proliferate via the oral tract of the host if propagules are ingested [14,25]. There is ongoing interest in the research of fungal entomopathogens as biological control agents for Arthropod pests [19,26,27]. Several species, such as *Beauveria bassiana*, *B. brongniartii*, *Lecanicillium lecanii*, *Metarhizium anisopliae*, *Isaria fumosoroseus*, and *Hirsutella thompsonii* are increasingly economically important in commercially developed mycoinsecticides [15,27,28,29,30].

The Asian paper wasp *Polistes chinensis* is one of five eusocial wasp species considered invasive in New Zealand [31,32,33]. It was accidentally introduced in 1979 and has since established a country-wide distribution [34,35]. With no native representatives of Vespidae in New Zealand, invasive eusocial wasps have little competition and have reached incredibly high densities in certain habitats [36,37]. These omnivorous wasps have catastrophic impacts on native invertebrate communities, and further disrupt ecosystems by competing for carbohydrate resources [38,39,40]. *Polistes* wasps in particular have been found to cause significant declines in butterfly populations, which can have cascading effects on plant productivity [41]. There is currently no efficient method for the control of paper wasp populations, and they are not attracted to the protein baits used for invasive *Vespula* wasps in New Zealand [31,42]. Options for biological control agents for *Polistes* species are currently the most promising avenue of research in this area [43].

During an ecological study of invasive *Polistes* paper wasps on Farewell Spit in New Zealand’s South Island, two morphologically distinct species of entomopathogenic fungi were discovered parasitising *Polistes chinensis.* The coastal saline vegetation abundant in this site has been identified as a key habitat area in which to study the ecological impacts of *Polistes chinensis* in New Zealand, as colonies reach the highest recorded densities in these habitats [44]. The site is also a national Nature Reserve [45] and Ramsar Wetland site [46], recently recognised for housing an impressive invertebrate diversity, including several rare and threatened species [47,48,49].

The aims of our study were: (1) to identify the two fungi morphospecies found infecting *Polistes chinensis* by combining genetic and morphological methods; (2) to test for the field prevalence of *Beauveria* sp. in samples of *P. chinensis* adults and larvae collected from wild nests; and (3) to test for disease causation in *P. chinensis* using samples of the identified fungi under controlled laboratory conditions.

## 2. Materials and Methods

### 2.1. Study Site, Sample and Nest Collection

Field work was conducted on Farewell Spit, New Zealand (40.513° S, 172.852° E) during the austral summer 2020–2021 and 2021–2022 seasons. Farewell Spit is a 25 km-long barrier sand spit extending east from the northern headland of New Zealand’s South Island [50]. It encompasses active and stable sand dunes, saltmarsh, and coastal scrub habitats [51,52]. The climate is temperate marine, with high relative humidity (yearly average 80.7%) and strong winds [53].

Locations of *Polistes chinensis* nests were established after visual searches in late November of 2020 and 2021 and monitored weekly through to late February of 2021 and 2022, respectively. With only three *Polistes* species currently present in New Zealand, the wasps were easily differentiated from congeneric species *P. dominula* and *P. humilis* by their colouration, particularly of the scutellum and second abdominal tergite, and patterns on the scutum [34,35,54]. Nests were primarily attached to low-growing, twiggy vegetation and rushes, the most common plant species being *Muehlenbeckia complexa*, *Coprosma acerosa*, *Ficinia nodosa*, *Coprosma tenuicaulis*, *Apodasmia similis*, and *Pteridium esculentum*, in order of frequency.

For molecular and morphological identification of fungi species, we collected dead and mycosed adult wasps on their nest combs (*n* = 17; Table S1). For analysis of fungi prevalence in the wasp population we collected healthy larvae (*n* = 12 nests) and adults (*n* = 8 nests) from monitored nests, with two individuals of each life stage from each nest. In the latter case, neither the larvae, the adults, nor their nests of origin showed signs of disease. All samples were either frozen or collected in 100% ethanol in the field and stored at −80 °C once in the laboratory.

For the laboratory infection bioassay, ten whole paper wasp nests were collected in mid-February of 2021 during daylight hours. Three of these nests had dead foundresses with presumed fungal infection (see Section 3.2; Table S1) affixed to the nest, as well as live adults. In two of these afflicted nests the emerged adult wasps had continued to construct the nest comb around the body of the foundress, so that it was partially covered in paper. The remaining seven nests were visually healthy, and were used in bioassay treatment and control groups. All nests were extracted by cutting away a small amount of the substrate vegetation and enclosing them in a ventilated plastic container. We remained at the nest sites up to half an hour after removing the nests to collect any returning adults. 

### 2.2. Fungal Identification

#### 2.2.1. DNA Extraction, Sequencing and Phylogenetics

We used four mycosed *P. chinensis* adult individuals for genetic identification of the infecting fungi, two per fungal species. For each fungal species, one sample was harvested from the outside of a frozen wasp cadaver, and one sample was harvested from the outside of a wasp cadaver stored in 100% ethanol. For each sample, we harvested 100 mg of conidia or fungal growth. The starting fungal material was collected in a 2 mL microtube (Sarstedt, Nümbrecht, Germany). We added three 3.2 mm stainless steel beads (Next Advance Inc., Troy, NY, USA), 1 mL of GENEzol DNA Plant Reagent (Geneaid Biotech, New Taipei City, Taiwan) and 5 µL of β-mercaptoethanol (Sigma Aldrich, St. Louis, MO, USA) to the tube. Samples were homogenised for two cycles of 10 s each at 10,000 rpm in a Precellys Evolution homogeniser (Bertin, Montigny-le-Bretonneux, France). We used a 24:1 chloroform–isoamyl alcohol mixture (BioUltra, Sigma Aldrich, St. Louis, MO, USA) to isolate DNA, followed by isopropanol precipitation (BioReagent, Sigma Aldrich, St. Louis, MO, USA), and a 70% ethanol purification step (VWR Chemicals, Poole, UK). Lastly, DNA was eluted in 75 µL of nuclease-free water (Ambion, Life Technologies, Waltham, MA, USA) and quantified using a NP80 NanoPhotometer (Implen, München, Germany).

Initially, we amplified two commonly used nuclear biomarkers for fungal identification, ribosomal DNA internal transcribed spacer (ITS) and ribosomal DNA small subunit 18S [55,56]. Primer pairs used were ITS1–ITS4 for ITS (Forward primer ITS4: 5′-TCCTCCGCTTATTGATATGC-3′ and reverse primer ITS1: 5′-TCCGTAGGTGAACCTGCGG-3′) and NS1–NS6 for 18S (Forward primer NS6: 5′-GCATCACAGACCTGTTATTGCCTC-3′ and reverse primer NS1: 5′-GTAGTCATATGCTTGTCTC-3′) from White et al. [57]. After the initial screening, one of the fungal species was identified as *Beauveria* sp. Thus, we also amplified the translation elongation factor 1-alpha gene (*ef1 α*) using primers developed for *Beauveria* species identification in New Zealand by McKinnon et al. [58] (see Section 2.3 below for primer sequences and further details). The reaction volume was 15 µL containing 1× MyTaq Red Mix (Bioline, NSW, Australia), 5 pmol of each primer, bovine albumin serum, fungal DNA, and nuclease-free water. Non-template controls were included in each PCR. We visualised PCR products by 2% agarose gel electrophoresis. Most PCR products were purified with rSap combined with Exo 1 (New England Biolabs, Ipswich, MA, USA). The *ef1 α* gene showed multiple bands, so the amplicon of the desired length was excised from the gel and purified with Zymoclean Gel DNA Recovery Kit (Zymo Research, Irvine, CA, USA). Sequencing was performed on an ABI 3130×1 Genetic Analyzer (Applied Biosystems, Waltham, MA, USA) at Massey Genome Service (Palmerston North, New Zealand).

Chromatograms were visualised, manually inspected for ambiguities and aligned using Geneious v. 10.2.6, Biomatters Ltd. (Auckland, New Zealand) [http://www.geneious.com, accessed on 6 October 2022] [59], with the default Geneious alignment algorithm. We manually trimmed the low-quality base calls at each end. We then blasted our fungal sequences into the BLASTn database in the NCBI website [60]. We searched against the rRNA/ITS databases. For ITS, we specifically searched the internal transcribed spacer region (ITS) from Fungi type and reference material. For 18S, we searched the 18S ribosomal RNA sequences (SSU) from Fungi type and reference material. For *ef1 α*, we searched the BLASTn standard database (default option). All sequences are archived in GenBank (accession numbers are presented in Table 1). 

To construct a phylogenetic tree for *Beauveria*, we downloaded the ITS sequences for the *Beauveria* species presented in Imoulan et al. [61] (GenBank accession numbers listed in Table 1 in Imoulan et al. [61]). An ITS sequence for *Cordyceps militaris* was also included as the outgroup. Sequences were aligned with Geneious with the default alignment algorithm, using global alignment with free ends and gaps and a cost matrix equal to 70% similarity. Clade probabilities were obtained from the posterior distribution using the MrBayes v.3.2.6 [62] plug-in for Geneious by Marc Suchard and the Geneious team (Auckland, New Zealand). Bayesian analyses were replicated twice, each with four Markov chains of 5 million generations, using a time-reversible model and gamma-distributed rate variation, with a proportion of invariant sites. Trees were sampled every 2500 generations, of which the first 150,000 generations were discarded as burn-in.

#### 2.2.2. Morphological Characterisation

**Figure 1:**
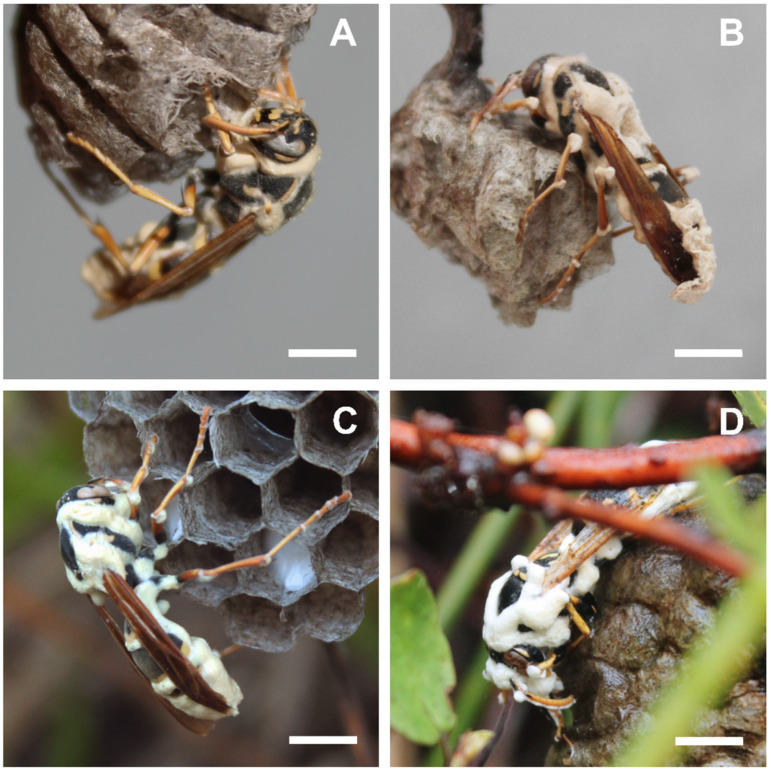
*Polistes chinensis* wasps infected with entomogenous fungus identified in this study as *Beauveria malawiensis*. These photos show host specimens on pre-emergent combs with no brood, although host nests were found in both pre-emergence and post-emergence phases. (**A**,**B**) Mycosed wasp cadavers on nest combs collected from the field on Farewell Spit, December 2020 and January 2021, respectively, photographs taken in the laboratory. (**C**,**D**) Mycosed wasp cadavers on nest combs in situ on Farewell Spit, January 2021 and February 2022, respectively. These specimens were discovered and collected at a mature stage of conidiogenesis, with significant external fungal bodies visible. Slight pinkish colouration of the fungal growth is evident in photo B, specifically on the antennae. Scale bars = ~0.5 cm. Photographs: Aiden Reason.

**Figure 2:**
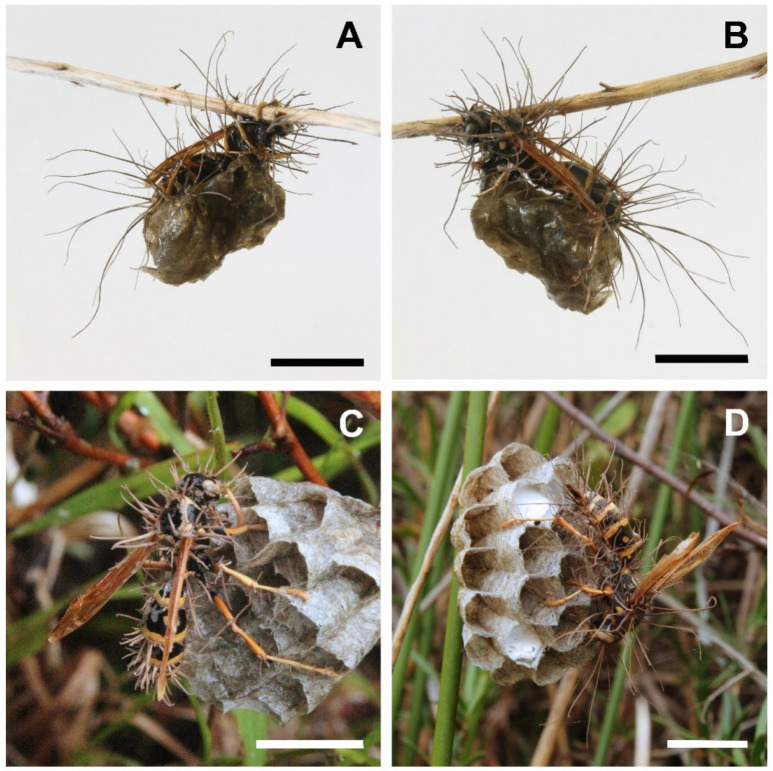
*Polistes chinensis* wasps infected with entomogenous fungus identified in this study as *Ophiocordyceps humbertii*. All specimens of this species were found on *P. chinensis* hosts affixed by their legs to the nest combs. These photos show host specimens on pre-emergent combs with no brood, although host nests were found in both pre-emergence and post-emergence phases. (**A**,**B**) A mycosed wasp cadaver on a waterlogged nest comb, collected from the field on Farewell Spit in December 2021, photograph taken in the laboratory. (**C**,**D**) Mycosed wasp cadavers on nest combs in situ on Farewell Spit, December 2021 and January 2022, respectively. Scale bars = ~1.0 cm. Photographs: Aiden Reason.

At the macroscopic level, the first fungal species had a pale, powdery appearance characteristic of the white muscardine disease caused by *Beauveria* species [63], emerging from between segments and joints in the wasps’ cuticle (Figure 1). Mycosed wasp specimens infected with the other fungus species developed white to brown, filamentous synnemata, emerging from the hosts’ intersegmental membranes (Figure 2), similar to descriptions for hirsutelloid *Ophiocordyceps* species [64,65].

For examination of fungal microscopic morphology, fungal samples were harvested from wasp cadavers under a dissecting microscope and mounted directly to microscope slides [66]. An Olympus CX41RF compound microscope with an Olympus DP22 digital camera was used to photograph and visually analyse samples.

### 2.3. Prevalence of Fungi in Wild Nests

We used the collected samples of individual adult wasps and larvae to test for presence of entomogenous fungi in apparently healthy individuals. Each larva was dissected, and their gut sac removed. The body remains were used for DNA extraction, for a total of 24 larvae from twelve different nests. We also extracted DNA from sixteen adult wasps from eight nests. DNA extraction followed the same protocols as described in Section 2.2.1 for molecular fungi identification.

To determine presence/absence of *Beauveria* spp. in these samples, we followed the nested PCR approach for identifying *Beauveria* species in New Zealand plants developed by McKinnon et al. [58]. Two-step nested PCR consists of standard PCR with primer pair 1, followed by standard PCR with primer pair 2. Briefly, a first primer pair, EF3F (5′-ACGGTGCCCGTCGGT-3′) and EF5R (5′-ACTTGATGAACTTGGGGTTGTTC-3′), amplifies 406 base-pairs (bp) of the *ef1 α* gene from multiple species of *Beauveria.* A second primer pair, EF4F (5′-GTCGCTGGTGACTCCAAGAA-3′) and EF4R (5′-GTACGGCGGTCGATCTTCTC-3′), amplifies a shorter fragment of the gene of approximately 200 bp, nested within the previous amplicon, and the resulting sequence contains positions where single nucleotide polymorphisms enable species identification [58]. The PCR reaction volume was 15 µL containing 1× MyTaq Red Mix, 5 pmol of each primer, bovine albumin serum, nuclease-free water, and 2 µL of wasp DNA (1 µL from each individual from the same nest). Non-template controls consisting of all the above reagents minus the DNA, and two independent samples positive for *Beauveria* extracted from infected adult *P. chinensis* wasps were included in each amplification assay. All PCR products were visualised by 2% agarose gel electrophoresis.

### 2.4. Infection Bioassays

Nests collected from the field were transported to the laboratory and transferred from collection containers into ventilated 9 L plastic, transparent boxes. Combs were secured by their supporting plant material to the box ceilings with wire. Eight nest boxes were used in total, with seven having one nest per box, and one with three nests in one “group” box. Three of the ten collected nests had a dead foundress affixed to the comb, presumably killed by fungal infection, but without evidence of conidiogenesis. Two of these infected nests were placed together with a third, apparently healthy nest, in the group box. The last infected nest was placed in its own box. These two nest boxes containing nests with infected foundresses were maintained free of bioassay manipulations solely for the purpose of monitoring the potential for infected foundresses cadavers to undergo conidiogenesis under increased humidity conditions, and potentially infect other wasps [15,66]. 

All the nest boxes were kept in a temperature-controlled rearing room set to 23.0 °C (varying with manual humidity changes) with an automated day-night light cycle of 12 h. Relative humidity was manually manipulated with five large water trays on elevated shelving and the use of two electric humidifiers (3.7 L and 5.6 L; Anko, Auckland, New Zealand), which were refilled daily. Both temperature and relative humidity were automatically logged hourly. Forager wasps were supplied with 30% sugar water, fresh water, cardboard paper for nest building, and wax moth larvae (*Galleria mellonella*) ad libitum. All wasps were allowed to acclimate for two weeks in the rearing room, during which time the average temperature was 23.8 °C (±SE 0.1), and the average relative humidity was 47.1 % (±SE 0.8).

A conidial suspension was prepared for each fungal species using the isolates harvested from the outside of host cadavers collected in the field. The conidia of the presumed *Beauveria* sp. were found to be highly hydrophobic and so were suspended in a solution of water with 0.05% Triton X-100 buffer (BioUltra, Merck, Auckland, New Zealand) [14] to improve suspension homogeneity. The concentration of conidia in this solution was measured using a Neubauer haemocytometer and determined to be 2.68 ×10^6^ cfu (colony forming units) mL^−1^ [66]. The synnemata isolated from the hirsutelloid fungus were suspended in water only, and the mixture gently agitated to release conidia. The conidia concentration of this solution was 1.52 × 10^6^ cfu mL^−1^. 

In a pilot experiment, we attempted host wasp infection using a spray method of inoculation with the conidia suspensions (and control solutions without conidia) as implemented in [66], but it did not result in any observable infectious effect. Thus, in this study, infection by oral route was attempted following methods outlined in Harris et al. [14]. The conidia suspensions were mixed 50:50 with 30% sugar water to be fed to treatment nests (*n* = 3). Similar solutions of 50:50 sugar water and the appropriate carrier per fungi treatment (pure water or water with 0.05% Triton X-100 buffer) were supplied to control nests (*n* = 3).

For initial exposure, three treatment nests were fed 0.8 mL of *Beauveria* sugar-conidia suspension, provided in shallow plastic dishes (3 cm in diameter). Three control nests were provided 0.8 mL of 50:50 sugar water and Triton X-100 solution in the same way. For the week following initial exposure, the temperature and relative humidity of the rearing room were maintained at averages of 23.6 °C (±SE < 0.1) and 46.0 % (±SE 0.6), respectively, consistent with the conditions of the acclimation period. After one week, exposure to the treatment and control solutions was repeated. At the same time, the humidity in the rearing room was increased, and maintained at an average of 66.8 % (±SE 0.9) for one week. We then continued to observe nests for 16 further days during which time relative humidity averaged 81.4 % (±SE 0.5), with average daily temperature of 21.5 °C (±SE 0.1).

The next assay consisted of two nests, one treatment and one control. One of these nests was provided 2 mL of 50:50 sugar water and conidia suspension of the second, unidentified fungus as described above (Section 2.4). The other nest was provided 2 mL of 50:50 sugar water and pure water as a control. For the following week, daily average humidity was 77.4 % (±SE 0.7) at 21.1 °C (±SE 0.1). The same feeding exposures were then repeated, and nests observed for the following eight days, with 63.5 % (±SE 1.0) average humidity and 21.7 °C (±SE 0.1) mean temperature. All nests were removed from nest boxes and all live and dead adults counted 45 days after beginning the *Beauveria* bioassay and stored at 4 °C.

We compared wasp mortality rates between treatment and control groups for each infection assay using a Kaplan–Meier survival analysis in R version 4.1.1 (R Core Team, Vienna, Austria) [67] with the *survival* and *survminer* packages [67,68,69]. The date of first exposure to conidia suspensions was set as day zero for each assay. 

## 3. Results

### 3.1. Fungi Identification

**Figure 3:**
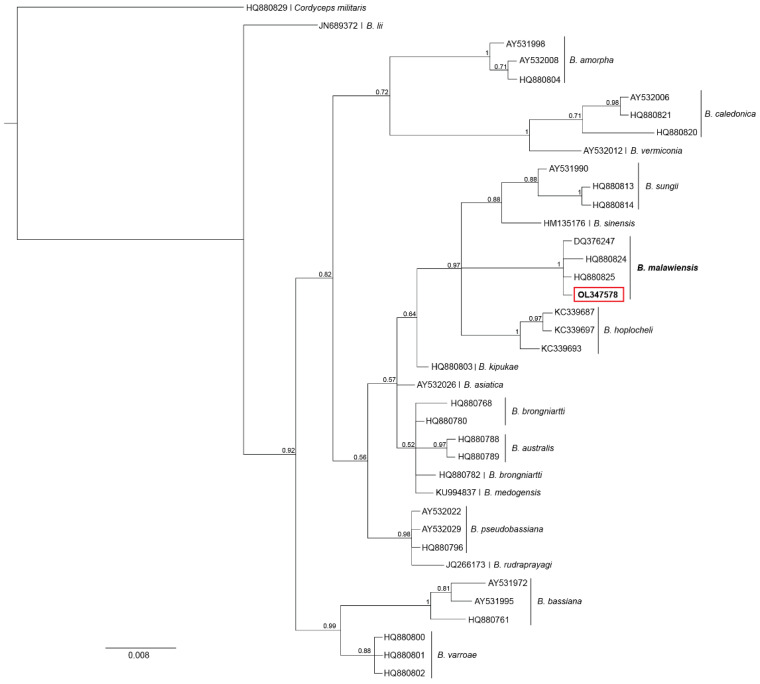
Bayesian phylogeny of *Beauveria* species based on the ribosomal DNA internal transcribed spacer (ITS) gene. Each individual isolate is shown by its GenBank accession number, and the species name noted. Posterior probabilities are presented above branches. The *P. chinensis* isolate generated in our study is in bold and inside the red box.

We found two species of fungi infecting *P. chinensis* paper wasps collected from Farewell Spit. Through molecular methods we were able to confirm that one of the fungi species belongs to the genus *Beauveria*. We amplified *Beauveria* isolated from two different mycosed wasps. For the first wasp, the resulting ITS sequence was of poor quality with significant noise (thus we did not submit it to GenBank), yet blasted to *Beauveria* sp. A sequence of 529 bp of 18S from this same wasp matched *B. caledonica* with 95.3% identical sites, and 178 bp of *ef1α* matched *B. malawiensis* with 100% similarity. The second *Beauveria* isolate resulted in 506 bp of good quality ITS sequence and matched *B. malawiensis* with 98.2% identical sites. For 18S, a 558 bp long sequence matched *B. caledonica* with 93.3% identical sites, and 178 bp of the *ef1α* gene matched *B. malawiensis* with 100% identical sites. The phylogenetic tree that includes many *Beauveria* species also confirm the identity of our isolate as *B. malawiensis* with a posterior probability of 1 (Figure 3). Considering the phylogenetic tree and the nested PCR protocol for *Beauveria* species identification developed by McKinnon et al. [58] combined, we can confirm that the *Beauveria* species found parasitising *P. chinensis* is *B. malawiensis*.

**Figure 4:**
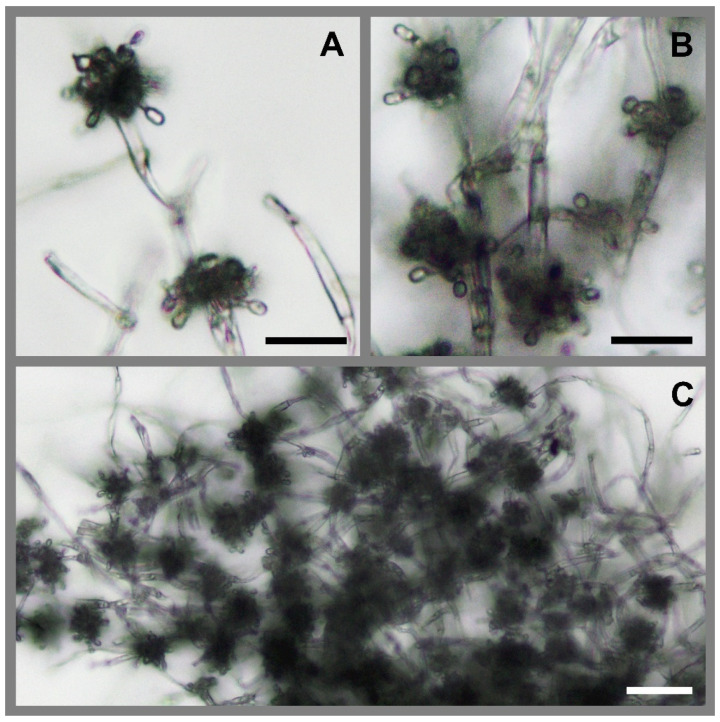
Microscope photographs of the fungal body from our wasps, which we identified as *Beauveria malawiensis*. The sample was isolated from a mycosed adult wasp infected in the laboratory and prepared without stain. (**A**,**B**) Globose to acutely obpyriform conidiophores with denticulate rachides and cylindrical conidia 3.3 × 1.6 µm on average. Scale bars = 10 µm; (**C**) Septate hyphae with condidogenous cells densely clustered. Scale bar = 20 µm. Photographs: Aiden Reason.

The morphological analysis of samples of *B. malawiensis* confirmed the molecular identification of this fungus, as the characters observed matched those described for the species [10,70,71]. *Beauveria malawiensis* samples had septate hyphae of 1.2–2.0 µm in width (average 1.6 µm, *n* = 20), occasionally branched. Conidiophores were globose to acutely obpyriform, with denticulate rachides and cylindrical conidia 3.0–3.7 × 1.2–2.1 µm in size (average 3.3 × 1.6 µm, *n* = 20; Figure 4).

DNA sequence data provided conflicting results for the hirsutelloid fungus. For the first isolate of this species, 266 bp of ITS sequence matched *Hirsutella citriformis* with 96.2% identical sites, and 566 bp of 18S matched the same species with 96.9% identical sites. The second isolate produced an ITS sequence of 394 bp that matched most closely to *Trichothecium crotocinigenum* at 96.6%, and an 18S sequence of 631 bp matched three different species all to 97.4% similarity: *Purpureocillium lilacinum*, *Metarhizium granulomatis*, and *Metarhizium viride*. Of all the conflicting genera matches that resulted from the different genes, only *Ophiocordyceps* spp.–revised nomenclature for *Hirsutella* [72,73]–provided a logical match to the general morphology and ecology of the fungus in question (see Figure 2). We built a phylogeny for our ITS and 18S isolates, but the alignments were poor, as previously reported for ribosomal DNA data for *Ophiocordyceps* [72,74]. The resulting unresolved tree is not presented.

**Figure 5:**
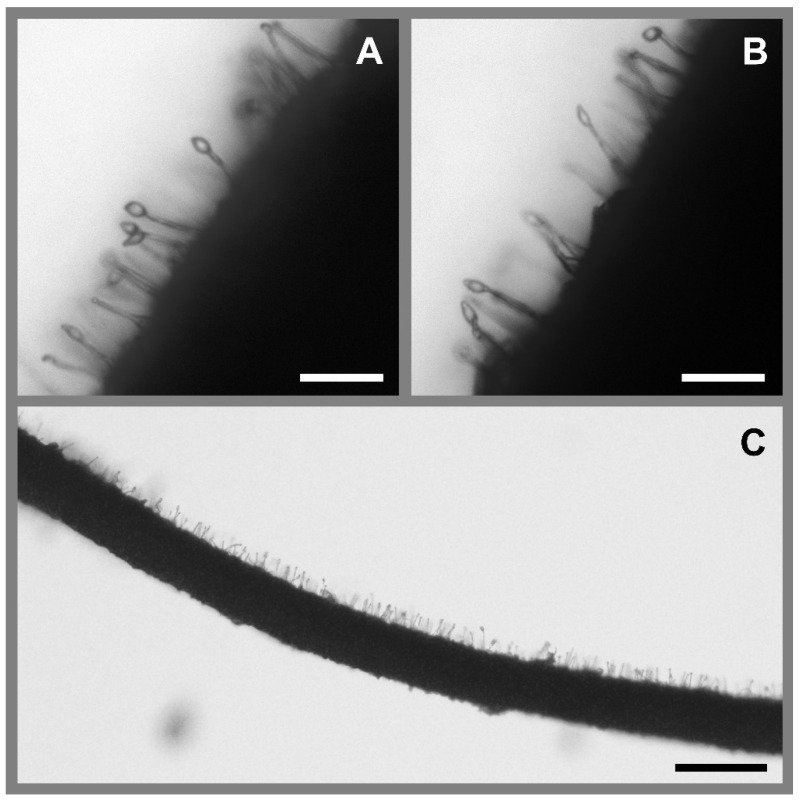
Microscope photographs of *Ophiocordyceps humbertii*. The sample was isolated from a mycosed adult wasp collected from the field and prepared without stain. (**A**,**B**) Conidiogenous cells with inflated bases tapering to slender necks, and allantoid conidia with mucilaginous coat 5.8 × 2.7 µm on average. Scale bars = 10 µm. (**C**) Conidiogenous cells arising laterally from a synnema, abundant on one side only potentially due to disruption during harvesting. Scale bar = 20 µm. Photographs: Aiden Reason.

Microscopic examination of isolated synnemata from this fungus confirmed presence of characters matching those described for hirsutelloid *Ophiocordyceps* spp. [75,76,77,78]. The mature synnemata of collected specimens were up to 12 mm long and 0.3–1.2 mm wide, arising from all over the host body at joints and inter-sclerite membranes, especially from between the thoracic and abdominal tergites. The synnemata were attenuated upwards and usually unbranched, white to brown, seemingly darkening with age. They were formed from longitudinal, densely packed septate hyphae 2.0–3.5 µm (average 2.7 µm, *n* = 20) in width. Conidiogenous cells arose laterally from the hyphae, uniformly dense to occasionally clustered. These ranged from 15 to 40 µm in length, basally inflated and narrowing abruptly to slender necks (Figure 5). Conidia had a mucilaginous coat, were hyaline and allantoid, and measured 3.5–7.1 × 2.2–4.3 µm (average 5.8 × 2.7 µm, *n* = 20). Due to the observed morphology and ecology of this species we putatively identify it as *Ophiocordyceps humbertii* (see Discussion Section 4). 

### 3.2. Prevalence of Fungi in Wild Nests

During the field study in the austral summer 2020–2021, a total of 495 *Polistes chinensis* nests were observed for fungal pathogens on Farewell Spit. Only seventeen nests (and one isolated wasp) were discovered with evidence of parasitisation by entomogenous fungi. Of these infections, three were identifiable as *B. malawiensis* and ten as *O. humbertii* (Table S1). The remaining five were unidentifiable due to having no external mycelial growth, yet could be recognised as internally mycosed. Diagnostic characteristics for an infection included the cadavers’ compound eyes and ocelli becoming paler brown or silver-coloured, and dullness of the wasp cuticle (Figure S1). Early stages of fungi emergence were often observed as the appearance of a fine white tomentum on the hosts’ antennae. In the following season, austral summer 2021–2022, 17 out of 522 recorded nests had evidence of fungal infection, with four identifiable as *Beauveria* spp., eight as *O. humbertii*, and five unidentifiable.

In the molecular assay, we found no evidence of *B. malawiensis* in the 24 *P. chinensis* larvae from the 12 wild nests examined, nor in the 16 adult wasps from eight wild nests collected from Farewell Spit. The prevalence of *Beauveria* in wild, healthy individuals, detectable using this method and population sample size, was zero.

### 3.3. Infection Bioassays

**Figure 6:**
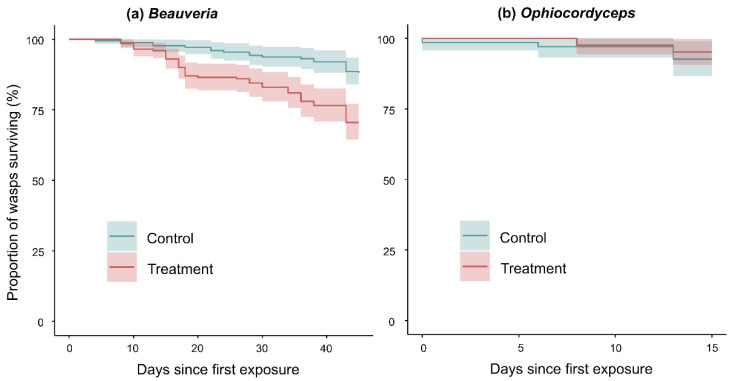
Kaplan–Meier survival plots showing the average (±95% CI) proportions of individual adult wasps surviving over time in each treatment group for each infection bioassay. (**a**) *Beauveria* assay; *n* = 3 nests per group. Survival probability begins to drop for treatment nests after the second exposure at day seven, when relative humidity in the rearing room was consistently above 65% and as high as >90%. Log-rank significance test for difference between groups: *p* < 0.001. (**b**) *Ophiocordyceps* assay; *n* = 1 nest per group. There was no difference in wasp survival rates between treatment and control nests, and no indication of positive infection observed (log-rank significance test: *p* < 0.511).

All three treatment nests in the *B. malawiensis* bioassay had an elevated mortality rate of adult wasps compared to controls (*p* < 0.001, Figure 6; Table 2). In two of these nests, conidiogenesis was observed from cadavers during the experiment (Table 2). Many cadavers from the third treatment nest, which had the highest number of dead adults at the end of the bioassay, later sporulated while in storage. Conidiogenesis was first observed in *B. malawiensis* treatment nests at day 16 after initial exposure. The treatment nest with the lowest mortality rate also had the lowest concentration of cfu per adult wasp due to a slightly larger adult population (Table 2). However, this was also the nest with the highest number of sporulating cadavers.

There was no significant effect of treatment in the *Ophiocordyceps* bioassay (*p* = 0.511, Figure 6). Both nests had the same mortality rate and no conidiogenesis of cadavers was observed.

In the two nest boxes containing nests with pre-infected foundress cadavers kept for observation only, there was no conidiogenesis observed, and no apparent evidence of infection of live adults. All nests in these boxes had adult mortality rates similar to control nests in applied bioassays.

## 4. Discussion

We identified two species of entomogenous fungi found infecting *Polistes chinensis* on Farewell Spit, New Zealand. 

The first species was identified through molecular methods as *Beauveria malawiensis*. This identification was confirmed by examination of key microscopic characters, especially the size and shape of conidia [10,70]. The known host range of *B. malawiensis* in New Zealand includes members of Coleoptera, Hemiptera, Hymenoptera, Orthoptera and Phasmatodea, with *Vespula* wasp species recorded as particularly frequent hosts [10,58].

Molecular analysis of the second fungal species yielded inconclusive results but provided a point of reference for further exploration. Molecular identification is inherently limited by the availability and quality of sequence data in reference databases [79,80,81]. This hurdle may be especially complicated for species with complex taxonomic nomenclature [82]. Following phylogenetic analysis and unification of dual nomenclature, *Hirsutella* species have been revised as hirsutelloid asexual morphs of *Ophiocordyceps* [73,83]. The distinctive morphology, microscopic characters, and ecology of this species strongly suggests the identity *Ophiocordyceps humbertii*, in the hirsutelloid form previously known as *H. saussurei,* the only *Hirsutella* species recorded from wasp hosts [11,72,75,77,84,85]. Similarly, *Ophiocordyceps humbertii* is the only hirsutelloid *Ophiocordyceps* known to infect Vespid wasps [77,86]. It was first described by Speare [84] as a parasite of *Polistes* spp., and is frequently reported infecting Polistine and Vespine wasps [11,75,77,78,87,88]. This fungi is recorded as an exotic species in New Zealand [89,90], previously known solely from Vespine hosts [11]. To our knowledge, this is the first record of *O. humbertii* infecting *Polistes* wasps in New Zealand.

The identification of *O. humbertii* can be further supported from observations by Somavilla et al. [87]. These authors noted pre-mortem behaviour manipulation of *Polistes* hosts by *O. humbertii* in Brazil. In species with nests initiated by a sole foundress (such as *P. chinensis*), mycosed individuals were observed having affixed themselves to nest combs, in both pre-emergence and post-emergence phase colonies, as we observed in this study. Only the asexual hirsutelloid form of the fungus was observed in these cases. The mucilaginous coat on *O. humbertii* conidia enables adherence to a new host upon contact [21,22,91]. Thus, manipulating host behaviour to induce conidiogenesis directly on a hosts’ nest likely increases the chances of contact with a new host. Somavilla et al. [87] hypothesised that these specimens may represent a cryptic lineage within *O. humbertii*, adapted for improved propagule dispersal in sole-founding social wasp species. During the field study we discovered two specimen wasp nests wherein this proposed strategy may have been successful, with infected cadavers of both the foundress wasp and an emerged adult present on the nest comb (Figure S2). Interestingly, we have also observed infected individuals of *P. dominula* in the Nelson region of New Zealand, exhibiting different pre-mortem behaviour consistent with that described for solitary wasps, and swarm-founding Polistine wasp species [87,88,92] (Figure S3).

The prevalence of the identified fungal species in the Farewell Spit paper wasp population is, from present evidence, very low. Nests with evidence of fungal infection constituted only about 3.3% of the total nests recorded in each season of the field study, with specimens identifiable as *B. malawiensis* and *O. humbertii* contributing 0.7% and 1.8% on average, respectively. Our attempt to experimentally detect potential asymptomatic carriers of *Beauveria* spp. in a sample of adult and immature individuals (*n* = 40) from this population yielded negative results. Given that this fungus was identified in only 0.7% of nests discovered in the field, it is likely that our sample size was too small for positive detection with the molecular method used. There may have also been other limitations to this assay, such as a negligible proportion of hosts exposed to *Beauveria* spp. remaining asymptomatic, or the time of sample collection being unideal for fungus propagation.

In the *B. malawiensis* infection assay, all three treatment nests exposed to the *B. malawiensis* conidia solution experienced significantly higher mortality rates than control nests. While there was no significant main effect of relative humidity on wasp mortality, a substantial increase in mortality rates for treatment nests beginning at approximately day 13 (Figure 6) coincided with an also substantial increase in humidity at the same time. This humidity spike was the result of a period of extreme wet weather locally. Temperatures between 20 and 30 °C and high relative humidity are generally required for the germination, hyphal growth, and sporulation of most fungi, including entomopathogens [15,16]. Exact thresholds vary, but some species may require relative humidity of up to 90% or more [15,16,66].

The concentration of *B. malawiensis* conidia in the sugar solution fed to treatment wasps was 2.68 × 10^6^ cfu mL^−1^. A set volume of 1.6 mL solution divided over two feedings per nest resulted in concentrations varying between treatment nests from 5.29 × 10^4^ to 7.52 × 10^4^ conidia per wasp. These concentrations fall within already determined successful ranges for pathogenicity in similar bioassays, of over 9.05 × 10^3^ cfu wasp^−1^ for *B. bassiana* and *B. malawiensis* in *Vespula* hosts [10,11,14], to 4 × 10^7^ cfu wasp^−1^ for *B. bassiana* in *P. dominula* larvae [12]. All the concentrations used for treatment nests in this study resulted in successful fungi infections, and some mycosed cadavers from each treatment nest sporulated. The resulting external fungal bodies were a morphological match to original samples. We consider that this experimental result satisfactorily confirms disease causation by the *B. malawiensis* samples collected from *Polistes chinensis* hosts on Farewell Spit. Previous infection assay studies have satisfied Koch’s postulates for microbial pathogenicity of *Beauveria* species to wasps in laboratory settings [10,11,12,14]. The present study differs in the husbandry of wasp colonies, which aimed to mimic field conditions as much as practical. Adults were kept with their natal nests and intact combs in relatively large (9 L) containers, with protein and carbohydrate food and nest-building resources provided. Thus, disease causality was confirmed in conditions closer to realistic colony dynamics than previously attempted in known studies.

In our *O. humbertii* bioassay, there was no significant effect of fungi treatment on wasp mortality, and no evidence of fungal infection was observed. There may have been many different factors that contributed to this result. However, we hypothesise that a key factor is that conidia of *O. humbertii* are unable to germinate and infect the host via the oral tract. While in other studies it has been shown that some *Beauveria* species can successfully colonise hosts inoculated via this delivery method, this ability is unusual for entomogenous fungi in Hypocreales [14,18,25]. As discussed in Section 4, it is more likely that *O. humbertii* conidia are dispersed by transfer from a mycosed wasp to a new host via direct contact [21,22,87,91]. In contrast, *Beauveria* colonisation of paper wasps in the field is more likely a result of contact with propagules away from the nest, on vegetation or via foraged prey [13,14].

*Polistes chinensis* is an invasive species in New Zealand. There is no effective landscape-scale control for this species currently available [31,42], although management options are under ongoing investigation [47]. Biological control is considered the most viable avenue for development to manage paper wasp populations [43]. The open-air nesting behaviour of *Polistes* makes these wasps more susceptible to predation or parasitism by other organisms, in comparison to ground-nesting wasps such as *Vespula* spp., for which previously introduced biocontrol agents have had non-significant population impacts [31,43].

As biological control agents, fungal entomopathogens are typically applied as inundative measures in a similar manner to a chemical pesticide [19,30,93]. This method contrasts with classical or inoculative biocontrol applications wherein there is an expectation for epizootic spread [11,15,20,30,94]. For inundative release, mass production of the chosen fungi is required, and there are several major constraints on this process. Although fungi of Hypocreales are usually more easily cultured than other entomogenous groups (such as Entomophthorales), the cultivation of many species is still difficult [15,20,95]. The formulation of a carrier material, solution, or other means of delivery must be properly suited to the infection pathways of the fungal agent. The delivery method must consider long-term propagule viability, relatively narrow environmental windows for completion of the fungi life cycle, and the level of host specificity [15,19,27,30,96]. While generalist entomopathogens such as most *Beauveria* species [16,97,98] are often used as biological control agents in agricultural or horticultural settings, they may not be suitable as biocontrol agents for invasive species in conservation contexts due to the potential for non-target effects. Species with narrow host ranges should be prioritised for further research and testing to minimise risks to non-target arthropod fauna [99,100,101], though there may be substantial diversity in different strains of the same fungal species [102,103]. Recent research suggests that some entomogenous fungi may prove viable candidates for further development as biocontrol agents of invasive *Polistes* wasps [12]. There is an interesting potential for further work in this area, particularly for relatively understudied species such as those identified in this study.

## Figures and Tables

**Table 1:** GenBank accession numbers for each fungal isolate sampled from four *Polistes chinensis* wasp hosts. We amplified three regions for the *Beauveria* species, identified as *B. malawiensis*: internal transcribed spacer (ITS), small ribosomal subunit 18S, and translation elongation factor 1-alpha (*ef1 α*). For the hirsutelloid species, identified as *Ophiocordyceps humbertii*, we amplified only ITS and 18S, as the elongation factor primers are *Beauveria*-specific.

Gene	Primer ref.	Fungal Species	Lab ID	GenBank Accession no.
ITS	[57]	*O. humbertii*	1	ON479659
			2	ON479660
		*B. malawiensis*	3	Not submitted
			4	OL347578
18S	[57]	*O. humbertii*	1	ON458754
			2	ON458755
		*B. malawiensis*	3	OL336513
			4	OL336514
ef1 a	[58]	*B. malawiensis*	3	OL348210
			4	OL348211

**Table 2:** Data for proportion of adult wasp deaths per nest over the course of each assay and concentrations of cfu (conidia forming units) per adult wasp, for nests in treatment groups. *n* = number of adult wasps per nest. *P_dead_* = the number of dead wasps as a proportion of the total. Days is the number of days since first exposure to conidia treatment. Conidiogenesis is defined as the visible emergence of mycelia from a dead, infected host.

Fungal Species	Nest ID	*n_total_*	*n_dead_*	*P_dead_*	Days	cfu Wasp^−1^	Conidiogenesis
*Beauveria malawiensis*	167	62	34	0.55	45	6.92 × 10^4^	No
	193	81	10	0.12	45	5.29 × 10^4^	Yes
*Ophiocordyceps humbertii*	277	57	57	0.26	45	7.52 × 10^4^	Yes
	293	83	4	0.05	15	7.33 × 10^4^	No

## Data Availability

The data presented in this study are openly available in the Supplementary Materials at https://www.mdpi.com/article/10.3390/insects13100922/s1. Novel fungal sequences generated in this study have been submitted to GenBank (GB) with the GB identifiers ON479659, ON479660, OL347578, ON458754, ON458755, OL336513, OL336514, OL348210, and OL348211.

## Supplementary Materials

The following supporting information can be downloaded at: https://www.mdpi.com/article/10.3390/insects13100922/s1, Figure S1: Development of conidiogenesis on *P. chinensis* cadavers over time; Figure S2: Potential disease transmission between nest mates; Figure S3: *Polistes dominula* cadaver with fungal infection; Data sheets S1, S2, S3: raw data on wild wasp colonies and infection assays; Table S1: Sample data for collected nests and individual wasp cadavers with evidence of entomogenous fungal infection.
