# Supplementary material for: Identity, Prevalence, and Pathogenicity of Entomopathogenic Fungi Infecting Invasive Polistes (Vespidae: Polistinae) Paper Wasps in New Zealand
Source: Insects. 2022 Oct 12;13(10):922. doi: 10.3390/insects13100922 (PMC9604194; doi:10.3390/insects13100922)
Supplement: Supplementary file 1 [file insects-13-00922-s001.zip › insects-1939931-Supplementary_material.pdf]

insects

Article

Identity, Prevalence, and Pathogenicity of Entomopathogenic Fungi Infecting Invasive *Polistes* (Vespidae: Polistinae) Paper Wasps in New Zealand

Aiden Reason *, Mariana Bulgarella and Philip J. Lester

School of Biological Sciences, Victoria University of Wellington, P.O. Box 600, Wellington 6140, New Zealand
* Correspondence: aiden.reason@vuw.ac.nz

Supplementary Materials:

Figures

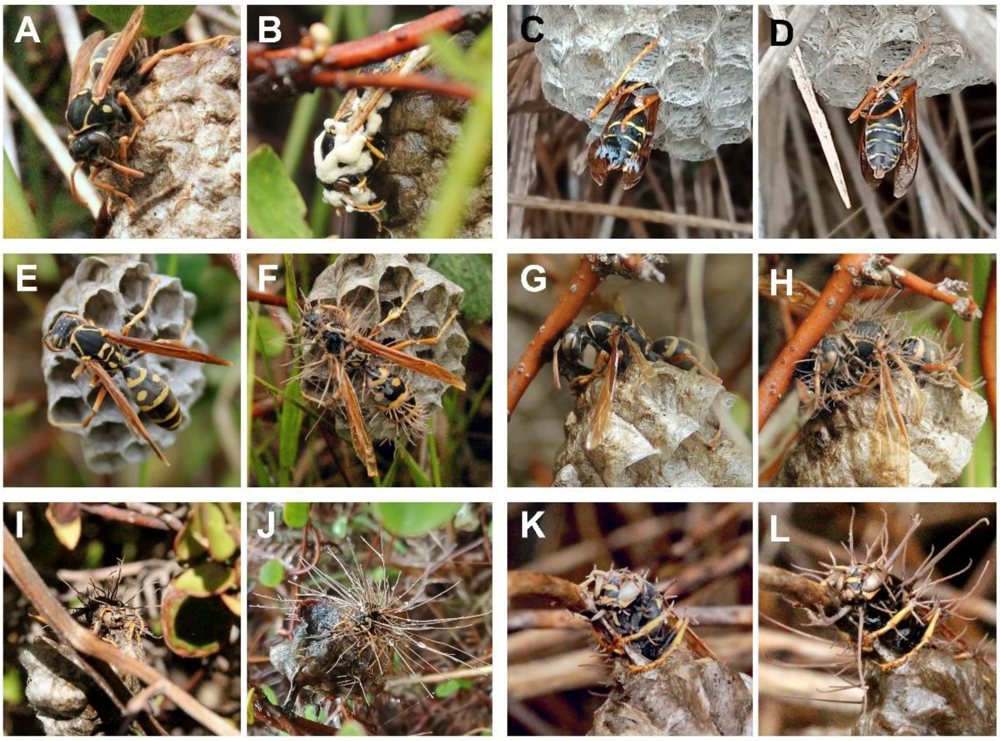

Figure S1. Development of conidiogenesis on *P. chinensis* cadavers over time. Six specimens of infected *P. chinensis* wasp cadavers with external conidiogenous bodies developing over time, photographed *in situ* on Farewell Spit over the 2021 – 2022 austral summer. Adjacent photographs are of the same mycosed specimen, with photos on the right side of each pair taken one to two weeks after the first. **A,B)** *B. malawiensis*, cadaver had pale ocelli and brown eyes in **A**, lightening slightly after conidiogenesis in **B**. **C,D)** *B. malawiensis*, having killed its host while the wasp was partially inside a comb cell; yellow colouration on the cadaver in **C** becoming dull in **D,** and white mycelium emerging from between abdominal sternites. **E – L)** *O. humbertii,* all host specimens with characteristically silver-coloured eyes and ocelli, and dull cuticle. **E,F)** and **G,H)** show development from having no external conidiogenous bodies to mature synnemata; **I,J)** and **K,L)** show development from young to mature synnemata. Host wasps are approximately 2 cm long. Photographs: Aiden Reason.

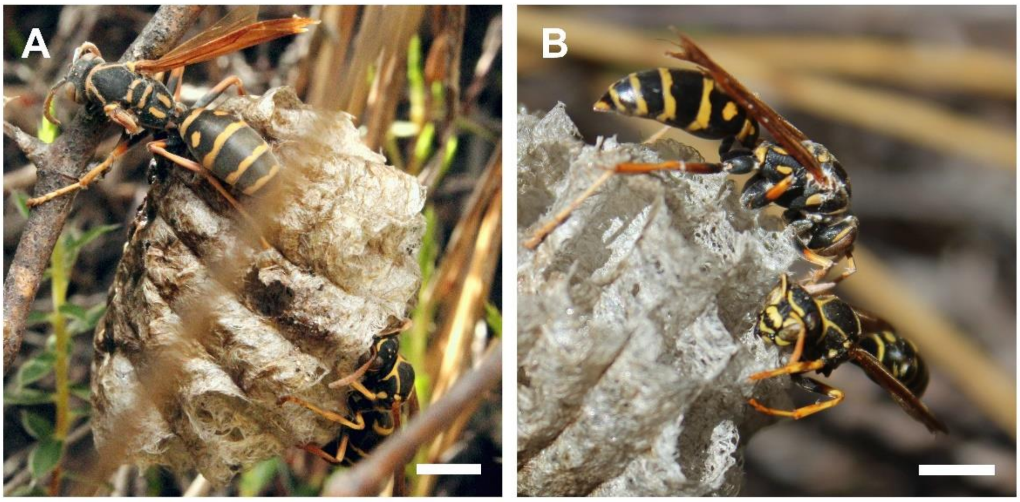

Figure S2. Potential disease transmission between nest mates. Specimens of *P. chinensis* nests wherein the foundress wasp is dead and infected with *O. humbertii*, and an emerged adult wasp cadaver on the same nest also shows evidence of fungal infection. It is possible that the daughter wasp in each case contracted their infections from the respective mycosed foundress, but we cannot confirm this. **(a)** This nest was discovered when the foundress (upper left) and daughter wasp (lower right) were both already dead and mycosed. The foundress cadaver was in the early stages of synnemata development. Photographed *in situ* on Farewell Spit, Jan 2022. **(b)** This nest was discovered in January 2021 with the foundress (upper left) dead and the cadaver in the early stages of synnemata development. At this time the daughter wasp was alive and appeared healthy. The nest also housed eggs, three larvae, and three capped pupal cells. We revisited the nest two weeks later, to find the daughter wasp dead with fungal infection, and the immature brood gone or undeveloped. Photographed *in situ* on Farewell Spit, Feb 2021. Photographs: Aiden Reason.

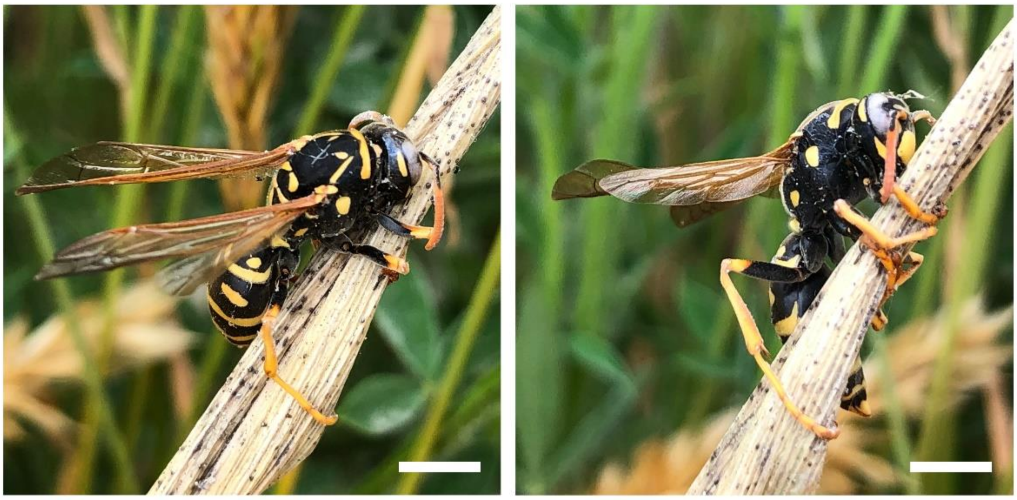

Figure S3. *Polistes dominula* cadaver with fungal infection, evidenced by silver-coloured eyes and ocelli, and a pale tomentum on the left antennae. The pose of the wasp body shows a firm grasp on a dry grass stem with the legs and mandibles, which is consistent with the pre-mortem behaviour observed from similar cadavers by Somavilla, *et al.* [1] and Sobzack *et al.* [2] in swarm-founding Polistine hosts. Photographed *in situ* in the Nelson region, New Zealand, Nov 2021. Scale bars = ~0.5 cm. Photographs: Mariana Bulgarella.

Tables

Table S1. Sample data for collected nests and individual wasp cadavers with evidence of entomogenous fungal infection. Where conidiogenesis was never observed in a specimen, fungi species is listed as “unknown.” Cells is the number of cells in the nest comb. Immatures includes larvae and capped cells with pupae.

Nest ID	Cells	Fungi sp.	Immatures	Live adults	Date found
254	32	*B. malawiensis*	-	-	12 Jan ‘21
367-B	*n/a*	“	-	-	10 Feb ‘21
BW1	17	“	-	-	18 Dec ‘20
241	20	*O. humbertii*	-	-	8 Jan ‘21
250	29	“	-	-	8 Jan ‘21
270	27	“	6	1*	15 Jan ‘21
271	18	“	-	-	15 Jan ‘21
273	28	“	-	-	15 Jan ‘21
275	15	“	-	-	15 Jan ‘21
326	15	“	-	-	4 Feb ‘21
346	10	“	-	-	5 Feb ‘21
379	29	“	-	-	12 Feb ‘21
SW1	10	“	-	-	18 Dec ‘20
136	35	unknown	-	-	**31 Dec ‘20
295	49	“	21	5	27 Jan ‘21
297	64	“	20	5	29 Jan ‘21
302	30	“	10	2	29 Jan ‘21
388	26	“	-	-	15 Jan ‘21

* Upon next visiting nest 270, this adult was dead on the nest also.

** Date that the foundress of this nest was found dead; the foundress was alive at previous visit on 17 Dec, with 12 immatures.

References

1. Somavilla, A.; Barbosa, B.C.; Prezoto, F.; Oliveira, M.L. Infection and behavior manipulation of social wasps (Vespidae: Polistinae) by *Ophiocordyceps humbertii* in Neotropical forests: new records of wasp-zombification by a fungus. *Stud. Neotrop. Fauna Environ.* **2020**, *55*, 23-28, doi:10.1080/01650521.2019.1691908.
2. Sobczak, J.; Somavilla, A. Manipulation of Wasp (Hymenoptera: Vespidae) Behavior by the Entomopathogenic Fungus *Ophiocordyceps humbertii* in the Atlantic Forest in Ceará, Brazil. *Entomol. News* **2020**, *129*, 98, doi:10.3157/021.129.0115.
